# Understanding the nature-wellbeing relationship in adults: a qualitative metasynthesis review

**DOI:** 10.1016/j.wss.2026.100359

**Published:** 2026-06

**Authors:** Jennifer E. van Bekkum, Craig W. McDougall, Charlotte Wendelboe-Nelson, Samantha S. Mason, Andrew James Williams, Ruth Jepson, Stephen Malden

**Affiliations:** a Scottish Collaboration for Public Health Research and Policy, University of Edinburgh, UK; b European Centre for Environment and Human Health, University of Exeter, UK; c OPENspace Research Centre, University of Edinburgh, UK

**Keywords:** Nature, Greenspace, Wellbeing, Relationship, Metasynthesis, Qualitative, Review, Health promotion

## Abstract

The natural environment is increasingly acknowledged as an important factor for influencing human health and wellbeing, with growing agreement among policymakers, researchers, and practitioners of its benefits. While nature access and quality are important considerations, wellbeing benefits may be more closely linked to individuals’ perceptions as opposed to the environmental characteristics. Due to a complex of personal and socio-cultural factors influencing the nature-wellbeing relationship, traditional linear models may not be sensitive enough to explore the nuanced, context dependent processes of how people relate to nature. To address this gap, we carried out a qualitative metasynthesis review of 49 studies exploring how direct experiences of living nature can impact human wellbeing for adults. Through iteration, interpretation and critique, the findings were organised into a theoretical framework. The review provides deeper and more nuanced understandings of the nature-wellbeing relationship than existing pathway models by unpacking how therapeutic nature encounters are formed through embodied sense-making, shifting attention from objective exposure to subjective experience. Findings highlight the importance of how we relate to nature through multisensory experiences and attunement. The review also demonstrates that the diverse wellbeing outcomes people experience from nature, both hedonic and eudemonic, can only be fully understood by acknowledging how individuals make meaning from their lived experiences, recognising that emotion-laden interpretation is central to human nature. The findings have both theoretical and practical relevance, highlighting the importance of: integrating experimental and more tacit forms of knowledge; relational values; and person-centred approaches to designing nature programmes and spaces.

## Research highlights

• Benefits from nature may be more closely aligned to individuals’ perceptions.
• Moving beyond linear models can capture complex, context-dependant understandings.
• Our qualitative metasynthesis explored the nature-wellbeing relationship.
• Findings highlight multisensory, emotion-laden and relational nature experiences.

## Introduction

The natural environment is widely recognised as a determinant of health (DEFRA, 2011), with growing consensus across policy, research, and practice about its positive impacts (Lovell et al., 2018). As urbanisation increases globally, with over 50 % of the population living in cities and projected to rise to 70 % by 2050 (United Nations Department of Economic and Social Affairs, 2018), access to nature is diminishing. Urban expansion driven by industrial and scientific advancements has reshaped cultural norms and lifestyles, distancing us from nature and encouraging its commodification as a resource (Richardson et al., 2022; Seto and Ramankutty, 2016). While modernity has improved health and social conditions through science and technology, it is also associated with materialism, individualism, consumerism and economic growth, which can harm our sense of wellbeing (Hanlon et al., 2012).

Mental health issues are now a leading global disease burden (GBD, 2019) worsened by the COVID-19 pandemic (Geary et al., 2021; WHO, 2022). Stress, anxiety and depression are rising, with the World Health Organisation (WHO) declaring stress the health epidemic of the 21st century (WHO, 2017). The international prevalence of mental health disorders is typically under-reported and under diagnosed (Kohrt et al., 2025). Global figures published from available data in 2019 suggest that one in eight people experience mental health conditions, with anxiety and depression being the most common (GBD, 2019). In high income countries with more accurate reporting these figures are greater. For example, in England one in four people experience mental health problems annually (NHS England, 2016), with disadvantaged communities facing greater risks (Department of Health and Social Care, 2023). In the Global North, rising demands on healthcare systems and greater awareness of nature-based health benefits have driven interest in green social prescribing as a low-cost, community-based intervention (Bloomfield, 2017; Robinson and Breed, 2019). In addition, urban planners are increasingly considering how natural spaces can support wellbeing including questions of design and usage (Cameron et al., 2020; Marselle et al., 2014).

**Fig. 1:**
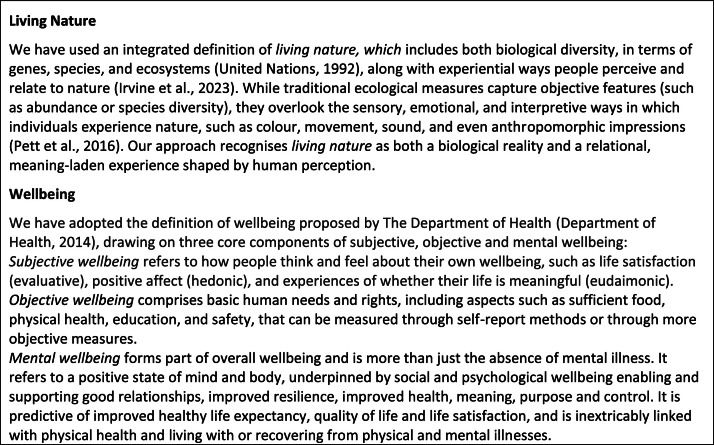
Definitions of living nature and wellbeing adopted for the study.

Mental health benefits of green spaces are well documented (Bratman et al., 2019; Hartig et al., 2014; Lovell et al., 2014) across different demographics (Richardson et al., 2019; Ward Thompson et al., 2012), but less is known about the specific therapeutic effects of contact with different types of living nature, such as plants, trees, animals and ecosystems (see Fig. 1 for full definitions). Spending time in and around nature-rich environments has been associated with higher self-reported happiness and wellbeing (Aerts et al., 2018; Cameron et al., 2020; Methorst et al., 2021).

However, improvements in wellbeing may be more closely linked to how individuals perceive biodiversity, rather than the actual nature present in an environment (Barnes et al., 2019; Cameron et al., 2020; Dallimer et al., 2012; Ma and Wu, 2025). While research in this area is still emergent and somewhat mixed, there is a growing consensus that peoples’ subjective perceptions of characteristics such as naturalness, multisensory attributes and safety align more closely with wellbeing outcomes than objective measures of biodiversity such as diversity and richness (Dallimer et al., 2012; Fisher et al., 2021; Irvine et al., 2023; Reyes-Riveros et al., 2021; Schebella et al., 2019).

This mismatch between objective measures of biodiversity and subjective perceptions has been termed the ‘person-biodiversity paradox’ (Pett et al., 2016) and highlights the importance of human experience, interpretation and perception in shaping responses to nature. This paradox is believed to be shaped by personal factors (e.g. characteristics, values, knowledge, experiences and nature connection) alongside embedded social and cultural norms, which can influence the magnitude and direction of a wellbeing outcome (Aerts et al., 2018; Dallimer et al., 2012; Pett et al., 2016, Farris et al., 2025). As a result, there is increasing interest in exploring this phenomenon from multiple disciplines to better understand the complex relationship between nature and wellbeing (Hedin et al., 2022; Marselle et al., 2021), as the underlying mechanisms remain relatively unchartered.

Research in this field commonly aligns with psycho-evolutionary theories such as Biophilia (Wilson, 1984), Attention Restoration Theory (Kaplan and Kaplan, 1989) and Stress Reduction Theory (Ulrich et al., 1991), which offer generalised explanations for our affinity with nature and its restorative effects. Concepts from ecological and environmental psychology such as nature connection (Mayer and Frantz, 2004) and affordances (Brymer et al., 2020) have advanced understandings by focusing on the dynamic relationship between people and their environments. In addition, theories and evidence aligned with social cohesion (Roszak, 2001; Weinstein et al., 2015), physical activity (Deci and Ryan, 1985b; Hoffmann, 1997; Koltyn, 1997), emotion (Bratman et al., 2021), physiology (Mills et al., 2017; Rook, 2013) and neuroscience (Sudimac and Kühn, 2022) all offer explanations that build on our knowledge of how nature impacts wellbeing in humans.

Marselle and colleagues (Marselle et al., 2021) developed a comprehensive pathway framework shaped from existing models (Bratman et al., 2019; Hartig et al., 2014; Markevych et al., 2017) outlining four ways biodiversity impacts health: reducing harm; restoring capacity; building capacity; and causing harm. To fully realise the wellbeing potential of nature, there is a growing recognition of the need to move beyond traditional linear models that may not be sensitive enough to explore the multifaceted, context-dependent mechanisms underpinning how people relate to living nature (Austen et al., 2021; Brymer and Davids, 2014; Harper et al., 2021; Robinson et al., 2024; Marselle et al., 2021). This review aims to address this gap by undertaking a qualitative metasynthesis that explores how direct experiences of living nature for adults can impact human wellbeing. Through unpacking individual experiences and identifying generative processes, we hope to add more holistic and complementary understandings to existing pathway knowledge.

## Methods

The study was registered with the International Prospective Register of Systematic Reviews (PROSPERO) in March 2024 (CRD-42,024,529,959). We carried out a metasynthesis (Lachal et al., 2017), drawing on principles from Critical Interpretive Synthesis (CIS) (Dixon-Woods et al., 2006) and meta-ethnography (Noblit and Hare, 1988). Qualitative research is a valuable method for gaining new understandings into health and medicine (Pope and Mays, 2013; Smith, 2024; Whitley and Crawford, 2005) and was chosen for analysis because it provides detailed accounts and rich descriptions of people’s nature experiences and wellbeing. We employed this metasynthesis approach to accommodate a large set of qualitative data varying in conceptual and methodological characteristics, and for its focus on inductive reasoning, critical engagement, interpretation, and mid-range theory generation (Depraetere et al., 2021; Dixon-Woods et al., 2006). This approach aims to provide conceptual insights and transferrable understandings that can inform policy and practice in meaningful and contextually-sensitive ways (Thomas and Harden, 2008).

### Search strategy

The development of the search strategy was iterative involving consultations with an information specialist, experimentation and consideration of balancing sensitivity with specificity (Methley et al., 2014). To be inclusive of relevant research fields (e.g. psychology, medicine and environmental sciences), multidisciplinary search recommendations for human-nature research were followed (Howlett and Turner, 2024) using the terms ‘nature’ OR ‘natural’, ‘green space’ OR ‘greenspace’ AND ‘biodiversity’ OR ‘trees’. These were combined with the terms 'wellbeing' OR 'well-being', OR ‘mental health’ AND ‘qualitative’ OR ‘interview*’ OR ‘focus group*’. Alongside the term ‘qualitative’, we included the method-specific terms ‘interview*’ and ‘focus group*’ to increase the search sensitivity as these common qualitative components of mixed-methods research are not always explicitly indexed or described as qualitative.

**Fig. 2:**
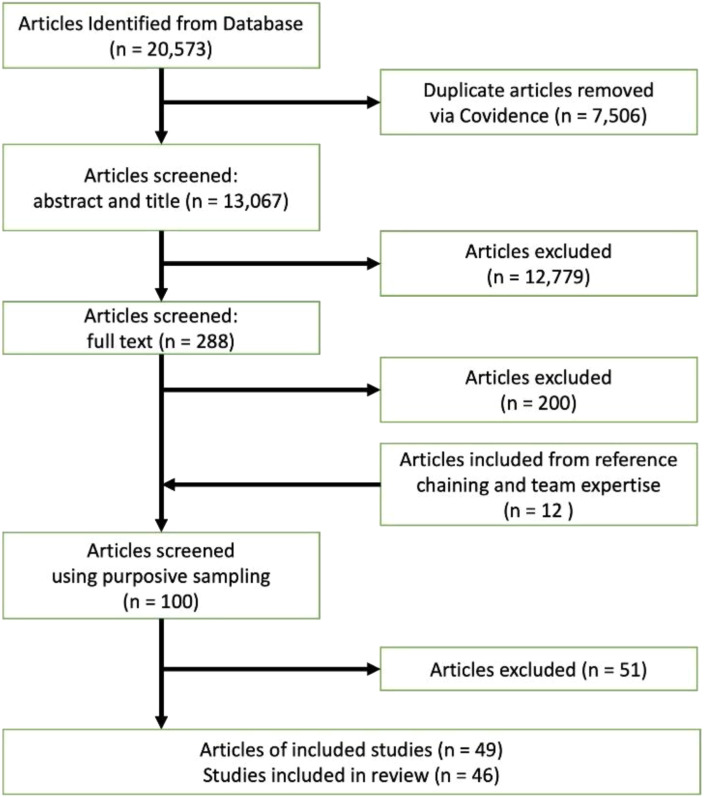
PRISMA flow diagram outlining the screening process for the final inclusion of articles in the metasynthesis review.

Systematic searches were carried out using electronic databases: MEDLINE, CAB, Embase, PsychInfo, and Web of Science in March 2024 (supplementary file 3). After removing duplicates, 13,067 titles and abstracts were retrieved and uploaded to the Covidence online screening and data extraction tool (Innovation, 2023) (Fig. 2). All titles and abstracts were reviewed by the lead researcher with 20 % reviewed by a second reviewer, which provided acceptable agreement of *a* ≥ 80 % compliance rate using defined inclusion and exclusion criteria (supplementary file 1). Full text screening of 288 articles was carried out independently by two researchers and 88 articles were selected for inclusion in the review. Reference chaining and team expertise were used to identify an additional 12 relevant articles for inclusion.

### Sampling

A proportion of the eligible studies provided limited or superficial insights into the nature-wellbeing relationship. In-line with previous meta-syntheses (Dixon-Woods et al., 2006; Orr et al., 2016; Thomas and Harden, 2008) purposive sampling was used to narrow down the cohort of articles for inclusion. Included studies were required to make a meaningful contribution to conceptual advancement through rich, in-depth accounts that helped to understand the mechanisms, processes and relationships shaping the nature experience and wellbeing. This resulted in a final core set of 49 articles agreed upon by the team for inclusion in the review.

### Quality assessment

As the analytical aim of metasynthesis is theory development and not aggregation of effect estimation (Depraetere et al., 2021; Thomas and Harden, 2008), conceptual richness was privileged over strict adherence to particular methodological standards; however quality assessment was undertaken to protect against the inclusion of any ‘fatally flawed’ papers (Dixon-Woods et al., 2006).

We had limited our search to studies published in peer-reviewed journals or those that had undergone formal assessment such as theses (supplementary file 1); therefore, the Critical Appraisal Skills Programme **(**CASP) 10-question checklist (Critical Appraisal Skills Programme, 2018) was deemed an appropriate quality assessment tool on two grounds. First, it is an educational tool commonly recommended for appraising the quality of healthcare research in qualitative evidence synthesis (Long et al., 2020; Noyes et al., 2018)**.** Second**,** it aligns with metasynthesis appraisal questions, addressing: clarity of aims and objectives, appropriate research design, methodological transparency, clear and logical links between data and interpretations, and appropriateness of analysis (Dixon-Woods et al., 2006; Lachal et al., 2017). In our study, CASP was primarily used as a safeguard, providing a transparent profile of evidence quality to identify any critical methodological limitations. Although there was some quality variation across studies (supplementary file 2), all articles were included as none were found to exhibit fatal flaws.

### Data extraction

To provide contextual information about the studies included in the review (Lachal et al., 2017), descriptive information (e.g. methods, populations, activities, settings and outcomes) was extracted systematically (Table 1). For the analysis, articles were uploaded into NVivo 14 (QSR Iternational Pty Ltd, 2023) and a broad approach was used, including findings and discussion sections, as author narratives can provide insight into the construction and framing of the enquiry (Dixon-Woods et al., 2006).

**Table 1:** Summary of included empirical studies in the metasynthesis review.

Author	Location	Study objective	Participants	Population Characteristics	Overall method	User Status	Summary of wellbeing findings
Acott et al. (2022)	UK; coastal blue/green space	To understand the relational values and importance of coastal environments for people	41 adults; 23 F, 18 M; no age stated	Mixed socioeconomic sample; predominantly white	Thematic analysis; interviews and workshops	Frequent and occasional coastal visitors	Mental wellbeing through aesthetics connection, enjoyment and restoration
Andkjaer et al. (2021)	Denmark; natural outdoor setting	To understand how persons with mental or chronic physical health problems who are self-referred or GP referred experience nature through rehabilitation programmes	21 adults;14 F, 7 M; no age stated	GP patients and self-referral	Philosophical hermeneutics; focus groups and observations	Nature programme users (low and high levels of activity)	Increased perceived health and wellbeing; mental health group attracted to calm and sensory activities
Bandukda et al. (2020)	UK; parks and natural outdoor settings	To understand the needs for blind and partially blind people when engaging in outdoor leisure experiences	29 adults; 12 F, 17 M; ages 25–82	Blind and partially blind	Thematic analysis; interviews and focus groups	Park and outdoor space visitors	Positive feelings of relaxation, excitement, nature connection and happiness
Bass and Loeffler (2023)	Canada; parks and natural outdoor settings	To understand the influence of mindfulness practice in nature on mental wellbeing and environmental stewardship	10 adults; 9 F, 1 M; ages 26–60	Post-secondary education; full-time employment	Thematic analysis; interviews	Park and outdoor space visitors	Gender issues influenced women's experience; practicing mindfulness in nature influenced feelings of connection and stewardship
Bell et al. (2015)	UK; coastal blue/green space	To explore coastal local residents’ everyday experiences of blue and green space in relation to wellbeing	33 adults; 20 M, 13 F; ages 25–85	Urban residents; mixed household income; mixed employment status	Thematic analysis; Interviews	Local residents	Enduring connections to the local coastline and various therapeutic outcomes
Bell et al. (2018)	UK; coastal blue/green space	To explore coastal local residents’ everyday experiences of blue and green space in relation to wellbeing	33 adults; 20 M, 13 F; ages 25–85	Urban residents; mixed household income; employment, mixed employment status	Thematic analysis; interviews	Local residents	Personal experiences of wellbeing from wildlife encounters
Bentley et al. (2023)	UK; forest/woodland	To examine how smells experienced in woodlands contribute to wellbeing across four seasons	194 adults; balanced genders; ages 18–60	Mixed sociodemographic; a mix of urban and rural residents across UK regions; 20 % non-white British	Qualitative content analysis; focus groups	Local residents	Smells associated with multiple wellbeing domains, positively and negatively
Birch et al. (2020)	UK; parks and urban natural settings	To explore the value of urban nature for the mental health and wellbeing of young people	24 young people; 14 F, 10 M; ages 17–27	From areas of urban deprivation; 50 % white, 50 % BAME; Nine of 24 participants had mental health difficulties	Diffractive and thematic analysis; interviews and art-based workshops	Local residents	Wellbeing benefits of acceptance, sense of self, connection, and care, with the human and non-human world
Carrico (2017)	USA; parks and natural outdoor settings	To explore participants’ experiences of walk talk nature therapy compared to a traditional office psychotherapy to support mental health	3 adults; 2 F, 1 M; ages 18–65	Previous users of counselling services	Cross-case synthesis and thematic analysis; interviews, observations and journaling	Nature programme users and non-users	Personally expansive, positive and spontaneous wellbeing such as increased self-awareness, enhanced mood and nature connection, and reciprocity
Cheesbrough et al. (2019)	Canada; parks and natural outdoor settings	To explore the perceived health and wellbeing effects of access to natural parks in a city	33 adults; 18 F; 15 M; ages 23–87	Predominantly higher socio-economic status	Thematic analysis; interviews	Local residents	Positive impacts on individual health and wellbeing from a wholistic experience of nature
Church (2018)	USA; parks and urban natural settings	To explore how urban residents perceive urban nature, and how proximity to and scale of nature influences connection	42 adults; 26 F, 16 M; ages 18+	Majority educated; mid-high incomes; majority white	Thematic analysis; interviews	Local residents with ecological awareness	Improvements in nature connection
Cocks et al. (2012)	South Africa; forest/woodland	To explore the cultural, spiritual and emotional relationships with nature expressed by Xhosa people	22 people; mixed gender, majority F; ages 12–67 (min. mean age = 27)	Indigenous people from regional villages and towns	Thematic analysis; interviews and focus groups	Local residents	Natural landscapes and biodiversity link to nature-based religious beliefs and connections, encompassing identity and wellbeing
Crockett et al. (2022)	USA; natural outdoor settings	To understand motives and psychological outcomes of mountaineering	10 adults; mixed gender; ages 47–84	Experienced mountaineers	IPA; interviews, fieldnotes, and documents	Mountaineers	Wellbeing underpinned by autonomy, competence and relatedness
Doughty et al. (2023)	Netherlands; green/blue space natural urban and rural setting	To explore everyday interactions with nature during Covid 19 restrictions for maintenance wellbeing	30 adults; 21 F, 8 M, 1 NB; ages 23–67	University educated or students; majority born and raised in the Netherlands	Thematic analysis; interviews	Local residents and students	Emotional and sensory experiences in nature and a greater sense of place contribute to increased wellbeing
Evans et al. (2023)	Australia; forest/woodland	To explore experiences of mentally ill people who attended forest therapy alongside their existing treatments	10 adults; 6 F, 3 M; mean age = 45	Patients; mixed employment status	Thematic analysis; interviews and journals	Previous users of counselling services for mental illness with mental and physical conditions	Increased sense of safety, spiritual growth, including acceptance gratitude and general wellbeing
Evered (2016)	UK; parks and urban natural settings	To investigate how different urban public spaces impact recovery from mental health problems	8 adults; 6 F, 2 M; ages 18–59	Patients	Thematic analysis; interviews and workshops	Mental health service users	Different places and their social and symbolic meanings can be beneficial and detrimental at different points of recovery
Finlay et al. (2015)	Canada; green/blue space natural urban and rural setting	How does taking part in a walk and talk programme influence older adults’ nature-health relationship	27 adults; gender not stated; ages 65+	Mixed socioeconomic status; varying proximity to green space, accessibility and walkability	Thematic analysis; interviews	Local residents	Positive impacts on physical, mental, and social health in later life
Gittins et al. (2023)	UK; forest/woodland	To understand the impact of a woodland activity programme on personal wellbeing over time	24 adults; 13 F, 11 M; ages 18–64	Mixed employment status; health/social care referrals and self-referrals; mental and/or physical health conditions and support needs, e.g. addiction recovery	Thematic analysis; focus groups	Nature program users	Wellbeing benefits such as perspective shifts, positive self-appraisal, increased confidence, lifestyle changes, social connection and support
Hill et al. (2014)	Australia; Spain; Mexico; rainforest, coast and sea	To examine how nature-based tourists encounter nature	285 adults; mixed gender; ages unclear	UK and international tourists	Grounded theory; interviews and open response questionnaire questions	Tourists interested in nature	Enhanced wellbeing through feelings of wonder, connection, spiritual fulfilment and mental restoration.
Hinds (2011)	UK; outdoors	To explore how a 10-day wilderness trip impacted wellbeing and environmental perceptions	5 adults; 5 F, ages 17–25	British middleclass	IPA; interviews	Nature programme users	Positive experiences such as connection, aliveness, contemplativeness, self-discovery, confidence and wellbeing - some emotional experiences remained ineffable
Iqbal and Mansell (2021)	UK; parks and natural outdoor settings	To understand the role of nature engagement in wellbeing and mental health	7 adults; 7 F; ages 18–50	Mixed ethnic backgrounds; international university students	Thematic analysis; interviews	Persons with nature engagement experience	Mental health and wellbeing benefits such as enhanced decision-making, productivity, social relations, freedom and coping
Irvine et al. (2023)	UK; forest/woodland	To identify how people conceptualise biodiversity and its wellbeing impact	194 adults; balance of gender; ages 18+	Mixed socioeconomic groups; mixed white British and other ethnicities; urban and rural locations	Thematic analysis; focus groups	Selected publics	Physical, emotional, cognitive, social and spiritual wellbeing outcomes
Johansson et al. (2024)	Sweden; natural outdoor settings	To understand the role of wildlife on perceived psychological restoration	28 adults; 15 F, 13 M; ages 18–75	University employees from three regions	Thematic analysis; focus groups	People interested in nature	Psychological benefits through improving restoration, and reducing stress and perceived threats
Jorgensen et al. (2007)	UK; neighbourhood woodland and trees	To reveal perceptions of aesthetics, safety and underlying meanings of woodland	39 people; F/M split fairy even; ages 15+ (min. mean age = 40)	White ethnicity; mixed occupations	Thematic analysis; interviews	Local residents	Enhanced wellbeing through feelings of relaxation, contentment, nature connection, seasonal awareness, and community belonging, with concerns over safety
Lorentzen and Viken (2020)	Norway; outdoors	To identify how nature might improve female immigrants’ mental health status	14 adults; 14 F; ages 27–70	International sample residing in Norway; mixed education level; mixed employment status	Qualitative content analysis; interviews	Immigrants	Mental health benefits from mood enhancement, familiarisation with culture, social interactions and physical activity
Macaulay et al. (2022)	Australia; parks and urban natural settings	To understand how people engage within urban nature and the associated psychological outcomes	20 adults; 14 F, 6 M; ages 24–55	University staff	Thematic framework analysis; interviews	Local residents	Wellbeing benefits, including relaxation, psychological distance from stress, and restoration linked to mindful and accepting engagement
Noe and Stolte (2023)	New Zealand; urban green space	To explore the significance of neighbourhood parks and home gardens in the lives of urban residents.	21 adults; 16 F, 5 M; ages 28–73	Majority European heritage New Zealanders; majority home owners	IPA; interviews	Residents, restoration volunteers and nature/park users	Positive personal experiences of nature and physical, mental and social health and wellbeing
O'Brien (2005a)	UK; forest/woodland	To understand social and cultural values of woodlands and trees in both urban and rural areas	123 adults; mixed genders; ages 21+	Mixed socioeconomic status and ethnicity; groups include: mothers from an inner city area, a woodland group from a deprived urban area, and a walking group	Thematic analysis; focus groups	Community group members and residents	Feelings of wellbeing along with conflict and confusion over what is viewed as anti-social behaviour
O'Brien (2005b)	UK; forest/woodland	To examine people’s values for woodlands in England	32 adults; gender not stated; ages 20+	Groups include: mothers from an inner city area, and a woodland group from a deprived urban area	Thematic analysis; focus groups	Community group members	Feelings of wellbeing along with barriers to greens pace and the importance of childhood memories and use of place
O'Brien et al. (2014)	UK; forest/woodland	To explore peri‑urban woodlands and the impact on health and wellbeing.	49 adults; 31 F, 17 M; ages unclear	Majority working or retired; white ethnicity; variety of able bodied, disabled and health problems	Thematic analysis; focus groups	Community group members	Health and wellbeing increases through social connections with others, sensory benefits and seasonal change
Pool et al. (2023)	UK; green/blue space natural urban and rural setting	To examine wellbeing benefits of local green and blue spaces for older adults	8 adults; 5 F, 3 M; ages 50–75	Not stated	Thematic analysis; interviews	Community group members	Value of natural spaces and nature/place connection
Puhakka (2021)	Finland; natural outdoor settings	To examine wellbeing effects of nature on university students	47 adults; F 38, M 8; ages 19–33	Environmental Science, Biology, Forestry and Agriculture university students	Thematic analysis; thematic writing	Students	Improved wellbeing from physical activity, metal restoration, stress relief, social relationships and self-reflection
Puhakka (2023)	Finland; natural outdoor settings	To examine how an outdoor education course, including canoeing and hiking trips, affects wellbeing and nature connection	16 adults; 13 F; 3 M; ages not stated	Not stated	Thematic analysis; interviews	Course members	Wellbeing benefits from mastery, beauty and group social support
Ratcliffe et al. (2013)	UK; parks and natural outdoor settings	To understand bird sounds in relation to attention restoration and stress recovery in nature	20 adults; genders not stated; ages 22–74	Majority British, UK residents in suburban and urban areas	Thematic analysis; interviews	Local residents	Wellbeing benefits from stress recovery and attention restoration
Samangooei et al. (2023)	UK; natural outdoor settings	To explore relationships with nature and particularly time alone	60 adults; mixed genders; ages 19–80	Mixed ethnicity and socioeconomic status; mixed health status; international sample	Thematic analysis; interviews	Publics	Wellbeing benefits from rejuvenation, stress relief, reflective thoughts and solitude
Seator (2001)	USA; natural outdoor settings	To investigate the experience of nature	23 adults; 21 F, 2 M; ages 22–74	White ethnicity; USA born; majority professionals	Heuristic/thematic analysis; focus groups and observations	Publics with nature connection	Wellbeing improvements from psychological processes, spiritual states, peace, wholeness, being and oneness
Shrestha et al. (2021)	Ireland; parks and natural outdoor settings	To explore psychological experiences associated with a brief nature or urban walk	13 adults; 9 F, 3 M; ages 18+	University students	Thematic analysis; interviews	Students	Positive affective and cognitive effects such as being energised and vitalised
Skår (2010)	Norway; forest/woodland	To explore the existential human–nature relationship	27 adults; equal gender split; ages 18–72	Selection based on a variety of ages and life stages	Thematic analysis; interviews	Local residents	Dynamic, socially constructed and changeable experiences, alongside gender-based fear in nature
Siltanen (2024)	Finland; parks and urban natural settings	To explore the relationship between perceived biodiversity and wellbeing	12 adults; 9 F, 3 M; ages 25–56	University students and professionals	Thematic analysis; interviews	Publics with nature orientation	Restoration, stress reduction and spiritual wellbeing through multisensory experiences and seasonal change
Sonntag-Öström et al. (2015)	Sweden; forest/woodland	To investigate the metal effects of patients with exhaustion disorder from a forest rehabilitation programme	19 adults; 16 F, 3 M; ages 29–60	Majority on long-term sick leave; patients with exhaustion	Grounded theory; interviews	Nature programme users	Improved mental restoration, reflection and helped with coping processes
Trangsrud et al. (2020)	Norway; natural outdoor settings	To explore experiences of a nature and recovery for persons experiencing eating disorders	8 adults; 7 F, 1 M; ages 19–41	Students and/or employed; all enrolled in or completed higher degrees	IPA; interviews	People with experience of eating disorders with an interest in nature	Improvements in calmness, sensory engagement, embodiment and self-care in a non-judgmental space
Uphold (2022)	USA; natural outdoor setting	To explore how veterans’ experiences of adventure-based counselling impacts their mental health	5 adults; 2 F, 3 M; ages 38–74	White ethnicity; employed or retired	Phenomenological analysis; interviews	Nature programme users	Wellbeing benefits relating to sense of self and identity, improved interpersonal relationships, sense of future, impact of natural environment and symptom reduction
Vaeztavakoli et al. (2018)	Iran; green/blue space natural urban	to explore people’s physical, mental and social health related to visiting urban water canals	200 adults; 102 F, 98 M; ages 15–85	Variety of professions	Qualitative content analysis; interview questions	Local residents	Physical, psychological, and social health including rehabilitation, restoration, social life, and place identity
Wadsö and Håkansson (2023)	Sweden; parks and urban natural settings	To explore perceptions of green neighbourhood interactions and engagement in activities	8 adults; 4 F, 4 M; ages 34–71	Mixed socioeconomic characteristics; participants from low and high green neighbourhoods;	Qualitative content analysis; interviews	Local residents	Green neighbourhoods increased activities, stress relief and helped to experience balance
Weimann et al. (2019)	Sweden; parks and urban natural settings	To understand different pathways between green environments, wellbeing and health	16 adults; 8 F, 8 M; ages 26–70		Qualitative content analysis; interviews	Local residents with moderate to high activity levels	Nature may promote wellbeing via activity, social support, restoration and sensory stimulation
Windhorst and Williams (2015)	Canada; parks and natural outdoor settings	To explore natural places and benefits to university students’ mental health	12 adults; 7 F, 5 M; ages 18–24	University students	Thematic analysis; interviews	Persons with strong nature connection	Mental health benefits from familiar places and separation from everyday life enabling relaxation and self-reflection
Yan et al. (2024)	China; parks and urban natural settings	To explore the subjective understanding of urban tranquil area	50 youth and adults; 25 F, 25 M; ages 14–72 (mean age = 28)	Mixed education level; mixed employment status	Grounded theory; interviews	Publics	Positive perceptions of tranquillity, relaxation alongside a state of calmness, pleasantness and immersion
Zeller (2006)	USA; natural outdoor setting	To explore connections and restoration in nature	7 adults; 4 F, 3 M; ages 30+	Not stated	Thematic analysis; interviews	Persons with strong nature connection	Improved stress relief and life balance, a sense of belonging and broader connection, awe, wonder and spirituality
Zhang et al. (2024)	China; forest/woodland	To explore atmosphere and experiences with animals through forest bathing	12 adults; 9 F, 3 M; ages 21–35	Non-local individuals who had visited the park at least twice	Thematic analysis; interviews	National Park users	Immersive experiences from animal encounters that astonish and rouse emotions and connections; positive and negative

*F* = female; *M* = male; NB = non-binary; IPA = Interpretive Phenomenological Analysis**Analysis**.

**Fig. 3:**
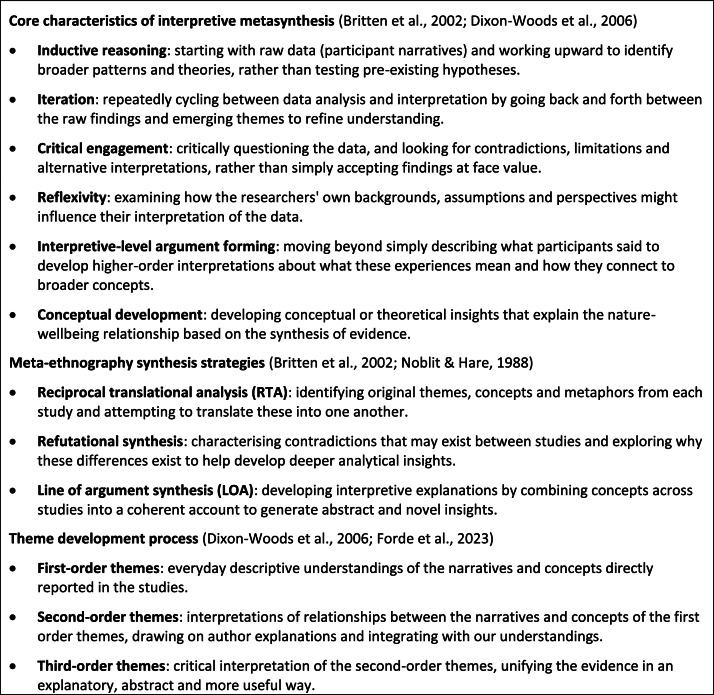
Definitions of analytical processes used in the metasynthesis.

The analysis focussed on inductive reasoning, iteration, critical engagement, reflexivity, interpretive-level argument forming and conceptual development (Lachal et al., 2017; Dixon-Woods et al., 2006; Britten et al., 2002) (Fig. 3). In line with the inductive nature of the analysis, research questions (supplementary file 1) were viewed as tentative guides as opposed to an anchor (Eakin and Mykhalovskiy, 2003) and subject to refinement through the research process (Greenhalgh et al., 2005).

The three meta-ethnography strategies (Fig. 3) of reciprocal translational analysis (RTA), refutational synthesis and lines-of-argument synthesis (LOA) were considered for use in the review (Britten et al., 2002; Noblit and Hare, 1988). Due to the size of the dataset, RTA was not deemed realistic or appropriate (Dixon-Woods et al., 2006). Refutational synthesis was incorporated throughout the analysis, attempting to explain contradictions and inconsistencies between studies and ideas. The final strategy of LOA was undertaken to develop interpretive third-order themes (Dixon-Woods et al., 2006), which was achieved by conducting a detailed analysis of the evidence, involving:

• Developing descriptive first-order themes through in-depth reading, notetaking and constant comparison that focussed on everyday understandings
• Clustering initial themes into groups and scrutinising their relationships
• Creating an explanatory second-order framework to present our understandings of the relationships at play
• Developing third-order themes through interpretation, critique, and refutation to go beyond existing explanations and to reconfigure these in a more abstract, powerful and explanatory way

Themes were carefully constructed through iterative engagement with the data (Fig. 3); however, due to the interconnected nature of many concepts, some overlap between themes reflects the complexity of the data rather than a lack of analytical rigour.

### Trustworthiness

**Fig. 4:**
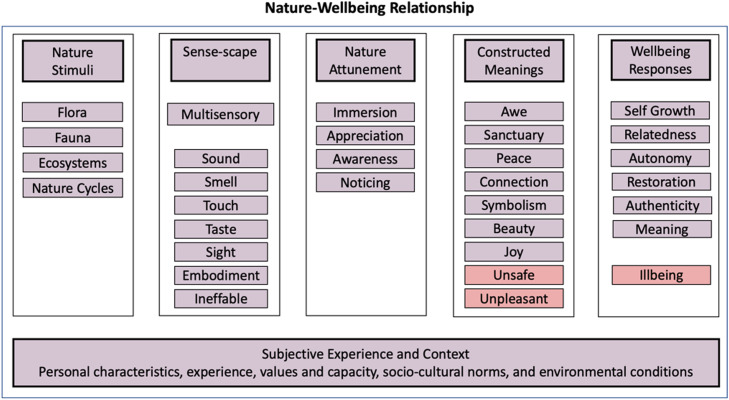
Framework outlining the elements identified within the nature-wellbeing relationship from initial data analysis.

Trustworthiness was supported through strategies such as LOA, independent coding of 12 % of the articles to help validate and refine themes, and team review of themes (Dixon-Woods et al., 2006). Reflexivity was enhanced by discussing the lead researcher’s views and staying close to participants’ voices using quotes. As interpretation shapes second and third-order themes, multidisciplinary input from the research team helped balance perspectives. To enhance transparency and value, we report data from first- and second-order themes (Fig. 4) to enable the reader to follow the interpretive process.

### Findings

Our review includes 49 articles published between 2001 and 2024, documenting qualitative studies and qualitative components of mixed-methods studies that examine adults’ direct contact with nature and wellbeing effects. These studies vary in their methodological approaches, participant populations and characteristics, types of nature activities, environmental settings and reported wellbeing (Table 1). The majority (92 %) of the studies included in the analysis are based in Global North countries, with the exception of four, situated in China (Yan et al., 2024; Zhang et al., 2024), Iran (Vaeztavakoli et al., 2018) and South Africa (Cocks et al., 2012). Only one study reports on an Indigenous population (Cocks et al., 2012). All themes were empirically established, supported by a substantial number of excerpts and rich narratives. Due to the large quantity of studies included in the metasynthesis, themes reported in the findings sections below are evidenced by selected quotes and citations only.

### Overview of initial themes: the nature-wellbeing relationship

To help make sense of the core elements involved in nature encounters, we first developed a visual model based on our inductive analysis (Fig. 4). The model outlines five interconnected elements identified within the nature–wellbeing relationship**:** nature stimuli**,** sense-scape**,** nature attunement**,** constructed meanings**,** and wellbeing responses**,** all underpinned by personal, socio-cultural, and environmental factors. While our visual framework offers a way to unpack these elements, we acknowledge it inevitably oversimplifies processes that are inherently dynamic, reciprocal, co-emergent, and non-linear.

### Subjective experience and context

**Fig. 5:**
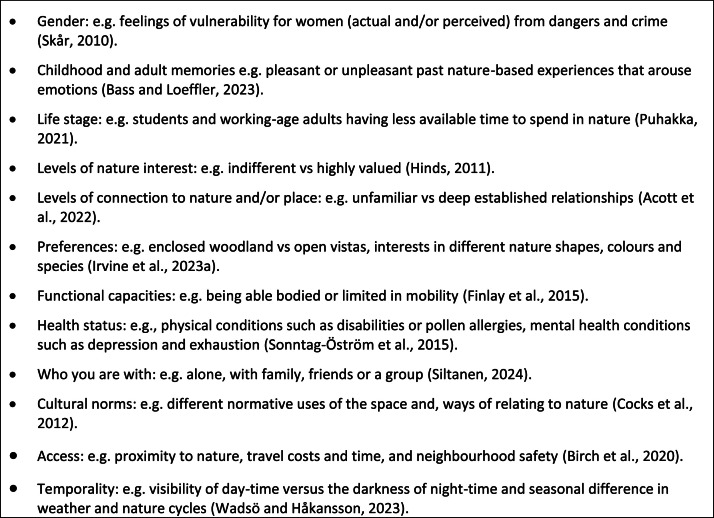
Examples from the review data of personal, social, cultural and environmental factors that impact the nature-wellbeing relationship.

Across the studies, it was found that a person’s subjective, lived experience was made up of their characteristics, histories, emotions, values, capacities, social norms and cultural backgrounds and played an integral role in how nature impacts wellbeing (Carr, 1970). Elements of subjective experience and wider environmental conditions (Fig. 5) were deeply interwoven throughout the proposed nature-wellbeing relationship framework, above.

### Nature stimuli

The descriptions of nature focused mainly on species and landscapes common to Europe and North America, with frequent mentions of trees, birds, forests, mammals and flowering plants. Less attention was given to insects, fish, fungi and reptiles. These natural elements were often described through their features, behaviours, life cycles, symbolism and even personality traits, such as squirrels being described as “*charming*” (Johansson et al., 2024) or trees as “*knotted with irregular branches*” (Abass and Serbeh, 2023). Emotional responses were strongly tied to these observations, such as “*The joy of hearing birdsong or watching trees rustle”* (Gittins et al., 2023).

While trees and birds collectively held particular significance, people’s preferences for plants and animals were deeply personal and shaped by cultural archetypes and meaning such as the excitement and awe of seeing a bird of prey (Johansson et al., 2024). Flora and fauna also represented living components of nature’s calendar cycles, embodying seasonal and temporal behaviours and growth patterns that signify the changing of the seasons, the passage of time and greater rhythms of life, linking to a more visceral, alternative understanding of time in relation to the natural world (Bell et al., 2018; Zeller, 2006).

Positive interactions with nature were found across different settings, from publicly accessible urban parks, waterways and neighbourhoods, to forests, rural parkland, countryside, outdoor wilderness settings and coastal areas. Preferences varied from managed to unmanaged, enclosed wooded areas to open expansive spaces with views, and from local pockets of nature in cities to remote untouched wilderness. The focus of the nature encounter was rarely on quantifiable characteristics such as abundance or diversity of vegetation but rather on the quality of living nature and characteristics that afforded feelings and emotions such as beauty, belonging or sanctuary for the individual, e.g*. “The trees, just gives you that sense of belonging, or just relaxing”* (Gittins et al., 2023).

### Sense-scape

The sense-scape represents the interface where internal self meets the sense-laden qualities of the external natural world. Visual aspects such as shapes, colours, patterns, movements along with smells sounds and on occasion taste, bound with personal emotions, messages, and bodily sensations were described, e.g. “*We don’t simply smell the leaf or the flower, we have a feeling about that perception that has the potential to be more subtle and complex… that says eat or don’t eat, move away or move close*” (Seator, 2000). The dynamic integration of sensory components could lead to immersive multisensory experiences. However, the sound of birdsong alone could evoke a sense of psychological sanctuary for someone who attached strong value to birds (Doughty et al., 2023).

Moving beyond the five senses, embodiment, referred to as haptic (Bell et al., 2015), was often experienced, e.g. “*A sense of being ‘grounded’ that reverberated through the body*” (Doughty et al., 2023). Going further still, experiences could become difficult to explain representing a deep and vivid sentiment of direct embodied communication beyond linguistic understanding (Bass and Loeffler, 2023), aligning with concepts such as affordances and atmospheres, rejecting the dualism between person and environment (Bell et al., 2023; Zhang et al., 2024).

### Nature attunement

The act of attuning to or noticing nature and the intensity in which this occurs was understood to impact the type of therapeutic effect a person can experience from nature (Crockett et al., 2022; Seator, 2001). The concept of attunement is discussed in profoundly qualitative and relational terms and moves beyond a conventional dose as quantified exposure time (Bratman et al., 2019; Richardson et al., 2021), emphasising a subjective and more experiential dose. Existing relationships with nature (e.g. identities, preferences and memories), alongside activities (e.g. sitting, walking and hiking), the company kept (alone, with family or a group) and the environmental stimuli (flora and/or fauna) played a role in the level of intensity in which people connected consciously and emotionally to nature.

A spectrum of nature attunement was apparent, at one end it may involve surface-level noticing of peace and quiet (Hinds, 2011) and at the other extreme it involves deep multisensory immersion (Bass and Loeffler, 2023; Bell et al., 2018), involving captivating feelings such as: being in the present moment; embodiment; a slowing down of time; being away; a shift in perspective to introspection and a more vital reality; and a clarity of mind or a spiritual connection to something greater, bringing with it emotions such as joy and awe, e.g. “*I am there in the moment. I am present, and I feel like I am absorbing all of the beauty around me. And I’m just taking it in—breathing it in. And I feel like it just nourishes me right through to my core*” (Zeller, 2006). While not all benefits of nature contact are enduring, more profound immersive attunement is understood to facilitate powerful psychological and spiritual therapeutic effects that can have a longstanding impact on a person’s sense of self and wellbeing (Mathers and Brymer, 2022).

We interpreted that immersive nature experiences can share qualities with contemplative practices such as meditation (Mathers and Brymer, 2022). Immersive experiences could be facilitated through structured outdoor mindfulness sessions (Evans et al., 2023) or occur spontaneously during organic encounters with nature (Johansson et al., 2024). More organic immersive moments may arise from slow-paced, contemplative experiences, such as walking or sitting quietly in nature (Seator, 2001), or from sudden, awe-inspiring moments such as witnessing rare wildlife (Hill et al., 2014). Traditional peak immersive experiences could be achieved through taking part in challenging outdoor activities (Crockett et al., 2022). However, not all immersive wildlife encounters were positive; some evoked discomfort or fear (Johansson et al., 2024).

The most profound immersive experiences reported were typically intimate, distraction-free, deeply sensory and introspective, often leading to personal revelations (Carrico, 2017; Iqbal and Mansell, 2021). Others described meaningful moments shared with trusted companions, especially those who held similar values or interests in nature (Cheesbrough et al., 2019; Siltanen, 2024). Some participants with greater experience or a long-standing interest in nature showed a heightened sensitivity and empathy to nature, treating it like a familiar companion (Crockett et al., 2022; Seator, 2001).

### Constructed meanings

The meanings people derive from nature are often immediate, emotional, and instinctive, shaped by their subjective experiences. These meanings are not simply based on what is physically present in nature, but on how the individual perceives and relates to it (Bell et al., 2018; Lorentzen and Viken, 2020; Puhakka, 2023). As a result, natural elements like trees or birds are experienced not just as objects, but as emotionally charged symbols or representations. The impact of the environment, therefore, is highly individualised, filtered through a person’s experiences, emotions and wider socio-cultural influences.

**Fig. 6:**
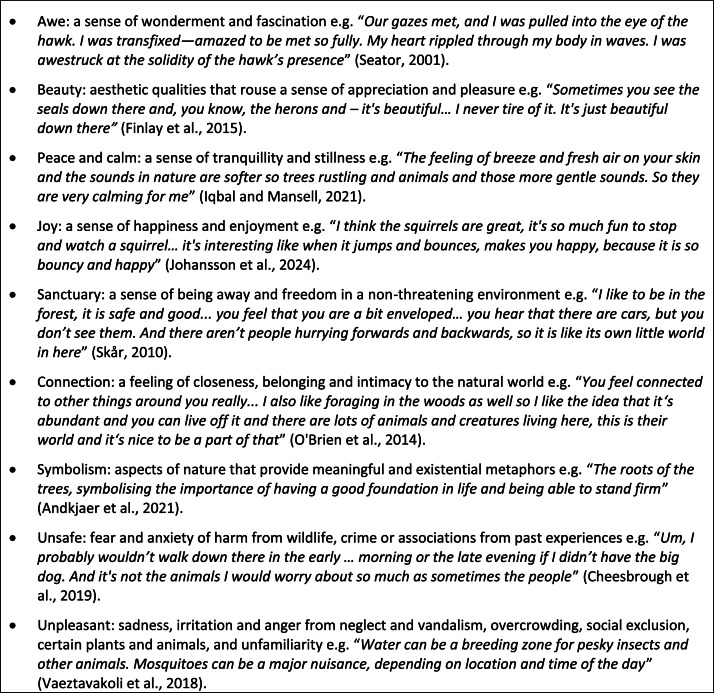
Example quotes of constructed meanings that arise from contact with nature.

A range of positive emotions were documented, such as: awe; beauty; peace; joy; symbolism; connection; and a sense of sanctuary (Fig. 6). Many of these emotions can be seen as expansive, playing a facilitative role in broadening the mind, leading to wellbeing responses in the form of emotional, cognitive, and spiritual growth, aligning with the broaden-and-build theory (Fredrickson, 2004). The feeling of sanctuary from everyday stressors and judgements (Iqbal and Mansell, 2021; Trangsrud et al., 2020) also provides another therapeutic function as people felt accepted in nature, mirroring the concept of ‘unconditional positive regard’ (Rogers, 1957), supporting a change in ones’ self-worth and leading to feelings of authenticity and autonomy.

While nature contact was found to bring positive emotions, it can also trigger negative feelings that disrupt therapeutic experiences (Cheesbrough et al., 2019; Lorentzen and Viken, 2020; Noe and Stolte, 2023; Vaeztavakoli et al., 2018). For example, fears of harm more commonly felt by women, sometimes fuelled by media narratives (O'Brien, 2005a) or real risks such as harassment (Jorgensen et al., 2007; O'Brien, 2005b), can hinder potential wellbeing benefits. Despite concerns, many valued their relationship with nature and negotiated these fears by joining groups, going out with family or dogs or using strategies like avoiding certain areas (Bass and Loeffler, 2023; Cheesbrough et al., 2019; Johansson et al., 2024). This negotiation was empowering for some and boosted self-esteem (Bass and Loeffler, 2023). In addition, some accounts discussed fear of wildlife (Hill et al., 2014; Johansson et al., 2024) and worries about slipping and falling (Hill et al., 2014; Pool et al., 2023). For a small group, time in nature could be anxiety-provoking, influenced by mental health issues (Sonntag-Öström et al., 2015) or personal histories, such as unfamiliarity with cultures, landscapes, or past experiences of war-related trauma (Lorentzen and Viken, 2020).

### Wellbeing responses

**Fig. 7:**
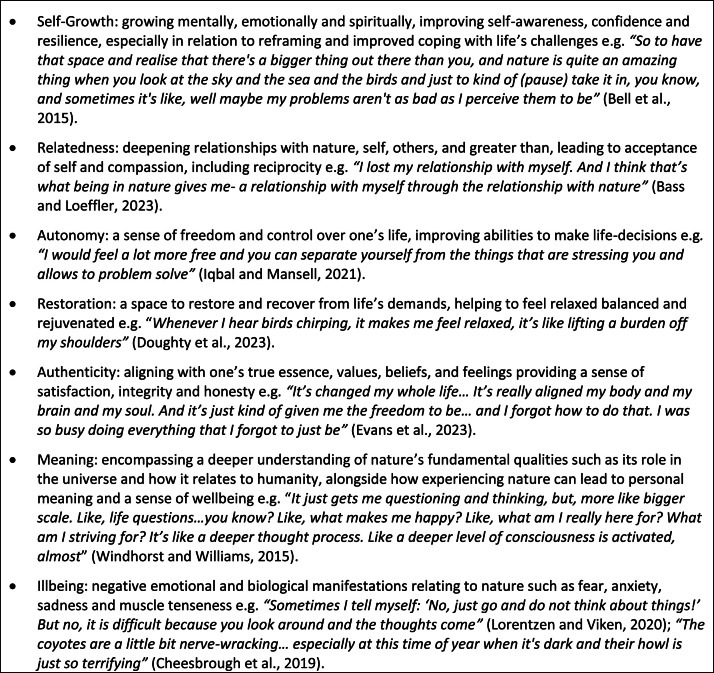
Example quotes of wellbeing and illbeing responses from contact with nature.

Therapeutic responses to nature can range from short-term and hedonic to more profound and eudaimonic. In the previous phase, positive emotional feelings (e.g. awe and beauty) were found to possess an expansive quality, opening space within the self to grow and develop, resulting in a host of wellbeing experiences grouped into broad concepts of: self-growth; relatedness; autonomy; restoration; authenticity; and meaning (Fig. 7). Feelings of restoration and wellbeing were not just experienced on a psychological level, but for some on a physical level too (Puhakka, 2021; Trangsrud et al., 2020), through biological systems linked to emotional regulation such as heart rate and blood pressure reduction (Bratman et al., 2021; Levine and al., 2021) and physical activity associated with the nature encounter (Lovell et al., 2014; Pretty, 2004).

Eudaimonic experiences often exhibited spiritual undertones relating to a broader sense of meaning and purpose in life, involving relief or letting go, acceptance and deep connection with self, nature or something greater (oneness) (Seator, 2001). The concept of relatedness also included realisations and feelings of compassion for nature and the wider ecosystems, deepening this relationship and developing a sense of reciprocity and care for nature (Carrico, 2017; Hill et al., 2014). Eudaimonic wellbeing was interpreted as aligning with the softening of ego and an expanding awareness of one’s true essence (Birch et al., 2020; Brymer et al., 2021; Seator, 2001). Negative emotions experienced by some, such as fear, loneliness or aversion (Lorentzen and Viken, 2020; Sonntag-Öström et al., 2015), if not supported appropriately, may contribute to illbeing responses in the form of stress, anxiety or depressive thoughts.

### Third-order themes

We present three third-order themes: the mechanism zone; the good life; and beneficiaries. These themes critically integrate core ideas from the wellbeing-nature relationship documented in the initial findings section (Fig. 4) and offer a more critical and abstract reconfiguration of the data.

### The mechanism zone

**Fig. 8:**
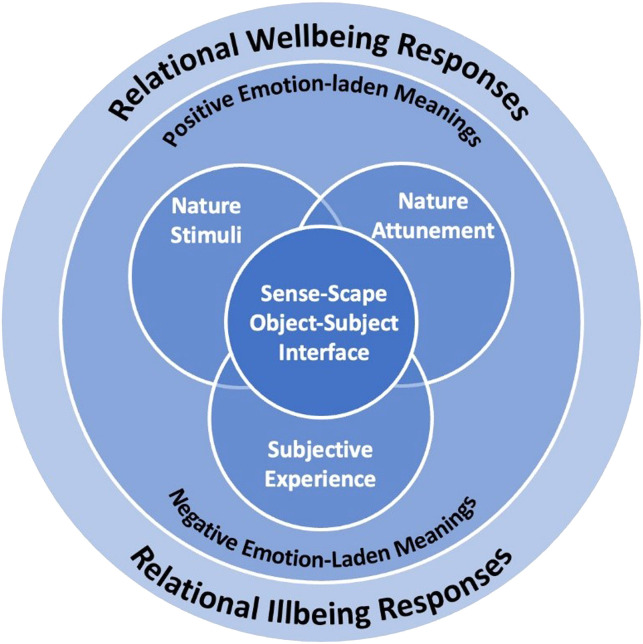
A nested model created from a relational values frame outlining the mechanism zone, comprising the sense-scape interface and the three core mechanisms of nature stimuli, subjective experience and nature attunement, which result in emotion-laden meanings and relational responses forming.

Drawing on the core elements presented in the nature-wellbeing pathway from the previous section, we used a relational values lens to frame how meaning is ascribed to nature and, in turn, how it impacts wellbeing (Pratson et al., 2023). We propose the concept of a mechanism zone, encompassing the interactive sense-scape where our subjectivity meets external reality (Fig. 8). Within this zone, we have identified three broad interdependent mechanisms that shape therapeutic outcomes:

1. Nature stimuli: material qualities of flora, fauna and ecosystems.
2. Subjective experience: representing how a person makes subjective sense of the world through personal characteristics, experiences, values, capacities and socio-cultural norms.
3. Nature attunement: representing an awareness, empathy and sensitivity to nature, varying in intensity like a dimmer switch (Dalkin et al., 2015), influenced by how you physically, cognitively, emotionally and socially engage with a natural setting.

These mechanisms co-create meanings rich in embodied emotion, such as feelings of beauty and awe (Fig. 6), that arise in the moment. These emotion-laden meanings can support deeper therapeutic relationships and discoveries described here as relational wellbeing responses (Fig. 7). Relational responses can be intrapersonal such as gaining perspective (Carrico, 2017; Gittins et al., 2023), and interpersonal such as feelings of connectedness with nature, others and spiritual dimensions (Hinds, 2011; Puhakka, 2021). We interpreted emotions sit at the core of the sensory interface (evidenced throughout the initial themes), and within the nature-wellbeing relationship can be understood as a type of felt knowledge in this experience-making space: forming bonds, communicating meaning and value, guiding morals, and forming identities or a sense of belonging. These interpretations align with existing constructivist social and psychological theories, which conceptualise emotions as forms of knowing (Barrett, 2017; Bevilacqua, 2023)

### The good life

The development of a positive relationship with nature can bring about feelings of hedonic and eudaimonic wellbeing (Siltanen, 2024). It has previously been proposed that attuning to nature fulfils a core psychological need and plays a role in living a worthwhile and happy life (Hurly and Walker, 2019; Richardson et al., 2021). The philosophical concept of ‘the good life’ aligns with eudaimonia, rooted in authenticity, a sense of purpose and meaning, relational values and virtuous living, amounting to human flourishing (Chan et al., 2018; Knippenberg et al., 2018; Ryff, 2023; van den Born et al., 2018). Not only can nature contact align us with the good life (Chan et al., 2016; Lima and Mariano, 2022), its capacity to provide a sense of judgment-free sanctuary can ameliorate ill-feelings and stressors stemming from modern socio-cultural conditions that prioritise individualism over community, competition over compassion, surplus over enough, and efficiency over meaning (Butler, 2019; Laruffa, 2022) .

Developing a relationship with non-human entities, such as animals, plants, trees and places that operate outside of cultural normative values and rules was interpreted as resonating with different parts of the self, heightening awareness, empathy and compassion (Stenfors et al., 2025; Yao et al., 2025). These experiences can result in encounters that go beyond momentary pleasure, developing a sense of personal growth, embodied connection, reciprocity and eudaimonic living (Bass and Loeffler, 2023; Seator, 2001). Being in nature can support the mind and body to take a break, enjoy being in the world in a different way, move past the outward facing societal mask or persona we wear to discover different, more essential parts of our self and psyche (Jung, 2014). Spending time in nature can shift individuals from an egocentric position to a greater awareness of biocentric values (Birch et al., 2020). This finding is supported in existing research (De Groot and Steg, 2007; De Groot and Steg, 2008; Mathers and Brymer, 2022) and highlights the therapeutic and transformative potential of moving beyond anthropocentric mindsets and conventional models of care. As definitions of health and wellbeing continue to expand (Burls, 2007; Engel, 1997; Irvine et al., 2019; Sulmasy, 2002), integrating more holistic perspectives into areas of healthcare, science, and society creates opportunities to consider what it means to live well and to recognise nature’s full capacity for fostering wellbeing.

### Beneficiaries

The elements outlined in the nature-wellbeing relationship (Fig. 4) are largely shaped by knowledge and values from the Global North, reflecting urban, affluent cultures with predominantly tamer ecosystems and anthropocentric assumptions such as viewing nature as an escape from modern life (Cheesbrough et al., 2019; O'Brien, 2005a). In contrast, the one Indigenous study included in the review was based in South Africa (Cocks et al., 2012) and exemplifies fundamentally different cultural conditions, relationships, beliefs and knowledge systems about nature, situating the literature from which understandings are developed, and its beneficiaries, largely within modern urbanised societies.

While nature offers wellbeing benefits, these are found to be most accessible to those with physical ability, financial resources, free-time, strong nature connection and the confidence to experience them (DEFRA, 2025), highlighting limited capabilities, resources, time and, particularly for women, safety concerns can restrict access (Bass and Loeffler, 2023; O'Brien, 2005b). Despite the numerous barriers for some, accounts demonstrate that people who value their relationships with nature actively try to negotiate the challenges faced to spend time in the outdoors (Cheesbrough et al., 2019; Johansson et al., 2024; Lorentzen and Viken, 2020). Although, those unfamiliar, ambivalent or adverse to nature were underrepresented in the data, it was interpreted that nature contact may be unfruitful or have negative consequences for wellbeing if not managed and supported adequately.

## Discussion

This qualitative metasynthesis review contributes to international conversations across research, policy and practice about the nature-wellbeing relationship. Our research included 49 diverse empirical studies that have not previously been synthesised in this way. Through the process of inductive reasoning, iteration, interpretation, and critique we have developed theoretical insights that deepen our understanding of how and why nature can improve people’s sense of wellbeing.

Existing pathway models have progressively advanced our understanding of how nature can impact human health and wellbeing. These models acknowledge the complex, dynamic qualities of nature encounters, providing a strong theoretical foundation (Bratman et al., 2019; Hartig et al., 2014; Markevych et al., 2017; Marselle et al., 2021). Cultural thirst for ‘credibility’ through objective measures (Greenhalgh et al., 2014) has resulted in current insights being largely informed by quantitative data underpinned by detached reasoning. This approach can sometimes privilege associations between measurable events and phenomena at the expense of understanding causal processes shaped by personal experience and socio-cultural contexts (Bratman et al., 2019; Harper et al., 2021).

Through the synthesis of qualitative evidence, our review complements existing pathway models in the field (Bratman et al., 2019; Marselle et al., 2021) by offering conceptual insights into how the elements of experience shape nature encounters and effect wellbeing. The methodological feature of ‘thick description’ (Geertz, 1973) has enabled an interpretive depth to the analysis that moves beyond observable and descriptive happenings to include explanation, meaning and context. In doing so, our metasynthesis has added ‘flesh to the bones’ (Miles and Huberman, 1994) of existing models by unpacking how environmental stimuli can take on therapeutic meaning through subjective, embodied and relational processes. Our work supports a growing body of research that proposes wellbeing responses align more closely with perceived than with actual nature (Dallimer et al., 2012; Fisher et al., 2021; Pett et al., 2016). It also complements similar realist review findings on green space interventions for improving mental health (Masterton et al., 2020) by providing a broader critical scope of everyday nature encounters.

The metasynthesis enabled us to identify and map out explanatory elements of the nature-wellbeing relationship (Fig. 4), highlighting the inseparable role of personal, socio-cultural, and environmental conditions in shaping the nature experience (Bell et al., 2023). Further analysis provided a more critical explanatory reconfiguration of the data, identifying three interpretive third-order themes.

The first theme outlines a relational mechanism zone, whereby experiences of nature emerge from the dynamic interaction between external stimuli, a person’s inner world, and their nature attunement. These interactions co-create emotional-laden meanings that shape therapeutic relationships with nature, self, and other (Fig. 8). A nested model was deemed a better fit than a linear model to depict the inseparable and reciprocal nature of stimulus-perception, objective-subjective relationships (Bell et al., 2023; Brymer et al., 2020).

While this model echoes ideas from ecological psychology, affordance theory and phenomenological dimensions, it focusses on the site of stimulus-perception as an emotive, relational space. In line with previous studies, we found sensory perception is far from a logical reflection of biophysical characteristics (Bell et al., 2023; Dallimer et al., 2012; Hartig et al., 2010; Wang et al., 2023), rather it presents as a deeply personal and dynamic process, value-laden from conception (Muraca, 2016). Therapeutic possibilities arise, in particular, from multisensory experiences (Bell et al., 2023; Franco et al., 2017; Harper et al., 2021; Kanelli et al., 2021) along with how we meaningfully attune to nature (Harper et al., 2021; Richardson et al., 2021; Richardson et al., 2016).

Existing research suggests that tuning into nature contributes to wellbeing beyond time spent in green space, indicating the importance of quality engagement (Richardson et al., 2021). We interpreted attunement in nature as an attentional hook into immersive encounters, rooted in awareness and empathy (Siegel, 2007). This reflects a deeper embodied type of engagement that can be facilitated in several ways through structured intervention (e.g. guided mindfulness practice), emergent nature encounters (e.g. noticing wildlife) or existing connection with nature.

The second theme aligns eudaimonic benefits from nature (McDougall et al., 2024) with the concept of the good life. In turn, it connects these benefits to the biopsychosocial-spiritual model of healthcare (Dyer, 2011), recognising that alongside our biological bodies, human presence and quality of life is also made up of emotions, meaning, contexts and relationships. While nature can offer a host of hedonic experiences such as a moment of peace or joy (Capaldi et al., 2014), the findings also indicate that nature encounters can lead to an array of eudaimonic wellbeing outcomes (Pritchard et al., 2020), such as personal growth and ego shifts leading to feelings of connection and the authentic self. Nevertheless, it is also important to note that in some instances, nature encounters can produce adverse effects and negative emotions (Bell et al., 2019; Marselle et al., 2021).

Eudaimonia and the good life relate to an ethical and existential agenda of how we live well, which is somewhat at odds with aspects of advanced capitalist culture, where economic growth imperatives and individualism contribute to psychological strain, loneliness and decreased life satisfaction (Daly, 2022; Noonan, 2024; Zhong et al., 2022). Nature in modern societies represents a sanctuary from the stressors of everyday life. It can be a physical or psychological place to escape, restore, relate or grow in a non-judgmental, essential way without social pressure, technology and everyday noise. Consequently, meaningful engagement with nature has the potential to provide affordable, large-scale preventative and restorative outcomes for conditions such as anxiety and stress, improving societal wellbeing, human flourishing, and stronger relationships with nature.

The third theme draws attention to the common beneficiaries of nature and the value frame from which the findings are situated. Along with much research in this field, we largely work from ‘truths’ underpinned by the dominant discourse of urbanised societies in the Global North (Kendal et al., 2020; Yang et al., 2021). In these contexts, those who report benefits from nature often have a pre-existing affinity for the natural world (Britton et al., 2020) alongside the required physical ability, time and resources to experience it in a positive way. Current understandings of nature and wellbeing provide limited insight into how people from different cultures, marginalised populations or who lack interest/experience relate to and construct meanings of nature encounters and, in turn, how they respond. To meaningfully address inequalities in the natural, social and health sciences, rather than making superficial gestures, we must fundamentally reconsider the Western-centric assumptions that shape our research methodologies, collaborative practices and institutional structures.

### Recommendations for policy and practice

In the context of rising global health challenges (GBD, 2019; Li et al., 2025; Wu et al., 2023), this review adds to current understandings about the therapeutic potential of nature contact. It supports the argument for more explicit recognition of wellbeing benefits humans can experience from nature within policy frameworks such as ecosystem services and nature-based solutions (Bratman et al., 2019; Marselle et al., 2021). Further, while policy discourse often frames nature in instrumental terms when addressing planetary or healthcare problems through stock-flow or provider-receiver models (Chan et al., 2018), this review proposes the value of a relational approach to understanding nature and wellbeing, addressing not only holistic human wellbeing but also the wellbeing of nature itself (Chan et al., 2016; Mayer and Frantz, 2004; Pritchard et al., 2020).

Relational values have more commonly been used to understand nature’s role within underrepresented communities such as Indigenous groups (Menzies et al., 2024; Urzedo and Robinson, 2023), however the inclusion of relational values is becoming more widespread in sustainability science and policy (Chan et al., 2016; IPBES, 2022; Pratson et al., 2023). This study supports the need for relational values to be further considered in public health and land management policies to better enable value-based, situational and cultural understandings of nature’s role for individuals and communities (Himes and Muraca, 2018).

Focussing on environmental factors alone, such as creating or protecting more green spaces and increasing access may be insufficient to encourage some people to spend time in nature (Lee et al., 2017; Lin et al., 2014), especially marginalised populations. While the environment plays an important role, the review demonstrates that we also need to pay serious attention to individual preferences and capacities alongside social norms and cultural sensitivities. This helps ensure that people identify with a space, feel safe and welcome, and have the societal 'permission' or motivation to be there (Wang et al., 2023). Research indicates this is especially pertinent for hard-to-reach communities, for example substance users may require more support (Masterton et al., 2022). Although largely out with the scope of this review, it is also worth noting the importance of social and pedagogical influences in supporting engagement with nature through programmes (Masterton et al., 2020; Mygind et al., 2019).

The review findings suggest that the concept of the nature dose understood in terms of frequency and exposure (White et al., 2019) should be expanded to include the type of encounter, how we attach personal and cultural meaning to it, and how we relate and attune to nature (Bell et al., 2019; Bratman et al., 2019; Richardson et al., 2021). This will improve understandings of wellbeing experienced across individuals, groups and cultures. Our study reinforces that nature can have a profound effect on more holistic wellbeing and flourishing. However, to optimise these benefits it is essential to design person-centred nature-based programmes and spaces that consider quality and appropriateness of the experience (Bell et al., 2019; Bloomfield, 2017). Such initiatives should be developed in collaboration with local communities to enable people to meaningfully engage and develop therapeutic relationships in and through nature.

### Strengths and limitations

The metasynthesis draws on established and systematic methodologies that apply epistemologically appropriate and developed processes to synthesise large sets of heterogenous qualitative data (Dixon-Woods et al., 2006; Noblit and Hare, 1988). The interpretive nature of the review implies an inherent level of authorial input. While, concerns have been raised about subjectivity leading to difficulties with transparency and reproducibility (Perlman et al., 2026), this should not be seen as a limitation from an interpretivist lens. Instead, it should be understood as part of constructivist knowledge, which is co-constructed responsibly and reflexively between the researchers and the data (Dixon-Woods et al., 2006).

Adopting a metasynthesis approach enabled conceptual innovation and the production of mid-range theories from diverse literature that can accommodate patterns across different contexts included in the review (Dixon-Woods et al., 2006; Noblit and Hare, 1988). This approach allows theories to be translated and interpreted responsibly with awareness of contextual biases. For example, as previously noted, the review data is largely situated in the Global North amongst general adult populations who have an existing degree of affinity with nature; therefore, in novel contexts, theoretical ideas will require further testing, refining and re-interpreting to ensure relevance.

### Further research

Future research should test and build on the findings from this review, particularly theoretical constructs around relational values and how people experience nature and wellbeing. More specifically, exploring how individuals meaningfully attune to nature could deepen our understanding of how to create and support therapeutic encounters. While multisensory experiences and prior nature connection have been found to facilitate attunement (Harper et al., 2021; Richardson et al., 2021), our research demonstrates complexities of this phenomenon that require unpacking further. In addition, socio-cultural barriers out with the scope of this review, such as ‘environment generational amnesia’ (Kahn and Weiss, 2017) and sensory and emotional dulling from phone use (Gao et al., 2023), may hinder attunement.

Further research is also needed to understand more about how positive emotions lead to eudaimonic wellbeing, and longer-term impacts. Studies suggest that positive emotion from nature contact can lead to long-lasting wellbeing (Bratman et al., 2019; Gittins et al., 2023) and that immersive nature-based experiences can also have a prolonged positive impact on people’s lives (Mathers and Brymer, 2022), but this requires further exploration. Crucially, future work must include underrepresented voices to ensure more equitable and informed public health and urban planning decisions.

## Conclusion

This review synthesised diverse qualitative literature into conceptual insights about the nature-wellbeing relationship by shifting focus from objective encounters to subjective experiences, helping to explain processes shaping therapeutic nature encounters. Rather than aggregating findings, our metanalysis offers contextually-grounded, meaning rich interpretations. Our study highlights that human encounters with nature are rarely experienced in quantifiable ways, supporting the use of a person-centred approach to designing therapeutic nature programmes and spaces.

## Declarations of the use of AI

During the preparation of this manuscript the authors used ChatGPT-4o**,** a model developed by OpenAI (2024), to aid the summarisation of some sentences and paragraphs, and to help with reducing word count. After using this tool, the authors reviewed and edited the content as needed, and take full responsibility for the content of the published article.

## Ethics statement

This metasynthesis review used data from previously published, publicly available studies. No new data involving human participants were collected and ethical approval was therefore not required. The study was conducted in accordance with the journal’s ethical standards and the principles of responsible research.

## CRediT authorship contribution statement

**Jennifer E. van Bekkum:** Writing – original draft, Visualization, Project administration, Methodology, Investigation, Funding acquisition, Formal analysis, Data curation, Conceptualization. **Craig W. McDougall:** Writing – review & editing, Visualization, Validation, Data curation, Conceptualization. **Charlotte Wendelboe-Nelson:** Writing – review & editing, Validation, Data curation. **Samantha S. Mason:** Writing – review & editing, Validation, Data curation. **Andrew James Williams:** Writing – review & editing, Supervision. **Ruth Jepson:** Writing – review & editing, Supervision, Funding acquisition, Data curation, Conceptualization. **Stephen Malden:** Writing – review & editing, Validation, Supervision, Methodology, Data curation, Conceptualization.

## Declaration of competing interest

The authors declare that they have no known competing financial interests or personal relationships that could have appeared to influence the work reported in this paper.

## Appendix Supplementary materials

Image, application 1

Image, application 2

Image, application 3
