# Supplementary material for: Understanding the nature-wellbeing relationship in adults: a qualitative metasynthesis review
Source: Wellbeing Space Soc. 2026 Jun;10:100359. doi: 10.1016/j.wss.2026.100359 (PMC13275795; doi:10.1016/j.wss.2026.100359)
Supplement: Supplementary file 1 [file mmc1.docx]

Supplementary file 1: Screening tool outlining inclusion and exclusion criteria

| **Screening Tool** | | | |
| --- | --- | --- | --- |
| **Title:** A Systematic Review of Qualitative Studies Exploring How Perceptions of Living Nature Impact Human Wellbeing | | | |
| **Research Question**   1. To explore the relationship that exists between perceptions of nature and wellbeing 2. To explore how subjective experiences and contextual factors shape the nature-wellbeing relationship | | | |
| **Population** | **Include** | **Exclude** | **Additional Notes** |
|  | Adult population 18 years and over (sample group mean must be 18 + for inclusion)  General population voices/users’ voices, general community | Studies including under 18-year-olds (sample group mean must be 18+)  Policy or professional voices e.g. farmers, nature programme leaders or guides, healthcare professionals administering programmes etc. | If they align with the study aims, including: Interventions, Indigenous studies, low-income countries, free-living older populations  Don’t include studies of non-free-living adults (e.g. nursing home residents, hospital in-patients)  Don’t include school/teacher focussed studies |
|  |  |  |  |
| **Intervention**  **(Research Focus)** | Studies that explicitly mention attributes of living nature (flora, fauna, ecological processes) | Virtual nature studies, indoor greenery, (not holistic) or greenspaces which are not free to access for all (not equitable) | If they align with the aims, include: blue space |
|  |  |  |  |
| **Comparators** | N/A |  |  |
|  |  |  |  |
| **Outcome** | At least one study theme to include perceptions of living nature  Wellbeing outcomes or pathway experiences (e.g. resilience, restoration, connection, peace, happiness, fear/anxiety) | Quantitative results  Mixed methods studies that don't include at least one theme on perceptions of living nature  Studies that do not include wellbeing outcomes | The focus is on data about participants experiences and perceptions of nature and how it impacts their wellbeing |
|  |  |  |  |
| **Study Design** | Qualitative studies (focus groups, Interviews, observational studies)  Mixed method studies that have a sufficiently qualitative element and depth analysis  Empirical peer-reviewed journal papers  Empirical dissertations and theses | Reviews  Non-empirical studies  Questionnaire/surveys  Grey literature | Don’t include surveys carried out via face-to-face interviews |
|  |  |  |  |
| **Other** | All dates  Multiple articles from the same study if offering novel insights | No dates excluded |  |
