# Supplementary material for: Understanding the nature-wellbeing relationship in adults: a qualitative metasynthesis review
Source: Wellbeing Space Soc. 2026 Jun;10:100359. doi: 10.1016/j.wss.2026.100359 (PMC13275795; doi:10.1016/j.wss.2026.100359)
Supplement: Supplementary file 2 [file mmc2.docx]

Supplementary File 2: Critical Appraisal Skills Programme (CASP) for articles included in the metasynthesis

| **Author** | **1. Was there a clear statement of the aims of the research?** | **2. Is a qualitative methodology appropriate?** | **3. Was the research design appropriate to address the aims of the research?** | **4. Was the recruitment strategy appropriate to the aims of the research?** | **5. Was the data collected in a way that addressed the research issue?** | **6. Has the relationship between researcher and participants been adequately considered?** | **7. Have ethical issues been taken into consideration?** | **8. Was the data analysis sufficiently rigorous?** | **9. Is there a clear statement of findings?** | **10. How valuable is the research?** |
| --- | --- | --- | --- | --- | --- | --- | --- | --- | --- | --- |
| Acott et al., 2022 | Yes | Yes | Yes | Yes | Yes | Can't tell | Yes | Yes | Yes | Yes |
| Andkjær et al., 2021 | Yes | Yes | Yes | Yes | Yes | Yes | Yes | Yes | Yes | Yes |
| Bandukda et al., 2020 | Yes | Yes | Yes | Can't tell | Yes | Can't tell | No | Yes | Yes | Yes |
| Bass & Loeffler, 2023 | Yes | Yes | Yes | Yes | Yes | Yes | Yes | Yes | Yes | Yes |
| Bell et al., 2015 | Yes | Yes | Yes | Yes | Yes | Can't tell | Yes | Yes | Yes | Yes |
| Bell et al., 2018 | Yes | Yes | Yes | Yes | Yes | Can't tell | Yes | Yes | Yes | Yes |
| Bentley et al., 2023 | No | Yes | Yes | Yes | Can't tell | Can't tell | Can't tell | Yes | Yes | Yes |
| Birch et al., 2020 | No | Can't tell | Yes | Yes | Can't tell | Can't tell | Can't tell | Yes | Yes | Yes |
| Carrico, 2019 | Yes | Yes | Yes | Yes | Yes | Can't tell | Yes | Yes | Yes | Yes |
| Cheesbrough et al., 2019 | Yes | Yes | Yes | Yes | Yes | Can't tell | Can't tell | Yes | Yes | Yes |
| Church, 2018 | Yes | Yes | Yes | Yes | Yes | No | Can't tell | Yes | Yes | Yes |
| Cocks et al., 2012 | Yes | Yes | Yes | Can't tell | Yes | Can't tell | Can't tell | Yes | Yes | Yes |
| Crockett et al., 2022 | Yes | Yes | Yes | Yes | Yes | Can't tell | Can't tell | Yes | Yes | Yes |
| Doughty et al., 2023 | Yes | Yes | Yes | Can't tell | Yes | No | Can't tell | Yes | Yes | Yes |
| Evans et al., 2023 | Yes | Yes | Yes | Yes | Yes | Can't tell | Can't tell | Yes | Yes | Yes |
| Evered, 2016 | Yes | Yes | Yes | Yes | Yes | Yes | Can't tell | Yes | Yes | Yes |
| Finlay et al., 2015 | Yes | Yes | Yes | Yes | Yes | Can't tell | Yes | Yes | Yes | Yes |
| Gittins et al., 2023 | Yes | Yes | Yes | Yes | Yes | Yes | Yes | Yes | Yes | Yes |
| Hill et al., 2014 | Yes | Yes | Yes | Can't tell | Yes | Can't tell | No | Yes | Yes | Yes |
| Hinds, 2011 | Yes | Yes | Yes | Can't tell | Yes | Yes | No | Yes | Yes | Yes |
| Iqbal & Mansell, 2021 | Yes | Yes | Yes | No | Yes | Can't tell | Can't tell | Yes | Yes | Yes |
| Irvine et al., 2023 | Yes | Yes | Yes | Yes | Yes | Can't tell | Can't tell | Yes | Yes | Yes |
| Johansson et al., 2024 | Yes | Yes | Yes | Yes | Yes | Can't tell | Can't tell | Yes | Yes | Yes |
| Jorgensen et al. 2007 | Yes | Yes | Yes | Yes | Yes | No | Cant tell | Can't tell | Yes | Yes |
| Lorentzen & Viken, 2020 | Yes | Yes | Yes | Yes | Yes | Can't tell | Can't tell | Yes | Yes | Yes |
| Macaulay, 2022 | Yes | Yes | Yes | Yes | Yes | No | Can't tell |  | Yes | Yes |
| Noe & Stolte, 2023 | Yes | Yes | Yes | Yes | Yes | Can't tell | Can't tell | Yes | Yes | Yes |
| O'Brien, 2005a | Yes | Yes | Yes | Can't tell | Yes (kind of!) | Can't tell | Can't tell | Yes | Yes | Yes |
| O'Brien, 2005b | Yes | Yes | Yes | Yes | Yes | Can't tell | Can't tell | Yes | Yes | Yes |
| O'Brien et al., 2014 | Yes | Yes | Yes | Yes | Yes | Can't tell | Can't tell | Yes | Yes | Yes |
| Pool et al., 2023 | Yes | Yes | Yes | Yes | Yes | No | Can't tell | Yes | Yes | Yes |
| Puhakka, 2021 | Yes | Yes | Yes | Yes | Yes | Can't tell | Can't tell | Yes | Yes | Yes |
| Puhakka, 2023 | Yes | Yes | Yes | Can't tell | Yes | Can't tell | Can't tell | Yes | Yes | Yes |
| Ratcliffe et al., 2013 | Yes | Yes | Yes | Yes | Yes | Can't tell | Can't tell | Yes | Yes | Yes |
| Samangooei et al., 2023 | Yes | Yes | Yes | Yes | Yes | Can't tell | Can't tell | Yes | Yes | Yes |
| Seator, 2000 | Yes | Yes | Yes | Yes | Yes | Yes | Yes | Yes | Yes | Yes |
| Shrestha et al., 2021 | Yes | Can't tell | Can't tell | Yes | Yes | No | Can't tell | Yes | Yes | Can't tell |
| Skar, 2010 | Yes | Yes | Yes | Yes | Yes | No | No | Yes | Yes | Yes |
| Slitanen, 2024 | Yes | Yes | Yes | Yes | Yes | Yes | Yes | Yes | Yes | Yes |
| Sonntag-Ostrom et al., 2015 | Yes | Yes | Yes | Can't tell | Yes | No | No | Yes | Yes | Yes |
| Trangsrud et al., 2020 | Yes | Yes | Yes | Yes | Yes | Yes | Yes | Yes | Yes | Yes |
| Uphold, 2023 | Yes | Yes | Yes | Yes | Yes | Yes | Yes | Yes | Yes | Yes |
| Vaeztavakoli et al., 2018 | Yes | Yes | Yes | Can't tell | Yes | Yes | No | Yes | Yes | Yes |
| Wadso & Hakansson, 2023 | Yes | Yes | Yes | Yes | Yes | Yes | Yes | Yes | Yes | Yes |
| Weimann et al., 2019 | Yes | Yes | Yes | Yes | Yes | No | Can't tell | Yes | Yes | Yes |
| Windhorst & Williams, 2015 | Yes | Yes | Yes | Yes | Yes | Yes | Can't tell | Yes | Yes | Yes |
| Yan et al., 2024 | Yes | Yes | Yes | Can't tell | Yes | Yes | Can't tell | Yes | Yes | Yes |
| Zeller, 2006 | Yes | Yes | Yes | Yes | Yes | Yes | Yes | Can't tell | Yes | Yes |
| Zhang et al., 2024 | Yes | Yes | yes | Yes | Yes | No | Can't tell | Yes | Yes | Yes |

Colours are used in the CASP appraisal table to provide objective insight about each study via a traffic light system (Yes = green; Can’t tell = orange; No = red)
