# Supplementary material for: Understanding the nature-wellbeing relationship in adults: a qualitative metasynthesis review
Source: Wellbeing Space Soc. 2026 Jun;10:100359. doi: 10.1016/j.wss.2026.100359 (PMC13275795; doi:10.1016/j.wss.2026.100359)
Supplement: Supplementary file 3 [file mmc3.docx]

Supplementary File 3: List of search terms used for each database

MEDLINE, CAB, Embase and PsychInfo (searched via OVID platform)

1. (Wellbeing or well being or mental health).mp. [mp=ti, ab, hw, tc, id, ot, tm, mf, bt, cc, tn, dm, dv, kf, fx, dq, nm, ox, px, rx, an, ui, sy, ux, mx]

2. (biodivers* or nature or greenspace* or green space*).mp. [mp=ti, ab, hw, tc, id, ot, tm, mf, bt, cc, tn, dm, dv, kf, fx, dq, nm, ox, px, rx, an, ui, sy, ux, mx]

3. (qualitative or interview* or focus group*).mp. [mp=ti, ab, hw, tc, id, ot, tm, mf, bt, cc, tn, dm, dv, kf, fx, dq, nm, ox, px, rx, an, ui, sy, ux, mx]

4. 1 and 2 and 3

Web of Science Core Collection (searched via Web of Science Platform)

"boidivers*" or "nature" or "greenspace" or "green space*" (All Fields) and "wellbeing" or "well being" or "mental health" (All Fields) and "qualitative" or "focus group*" or "interview*" (All Fields) and Review Article (Exclude – Document Types) and Book Chapters or Editorial Material (Exclude – Document Types) and Letter or Note (Exclude – Document Types)
